# Changes in audiovestibular handicap following treatment of vestibular schwannomas

**DOI:** 10.1017/S002221512300213X

**Published:** 2024-06

**Authors:** Tim Campbell, Shao Jie Goh, Andrea M Wadeson, Simon R Freeman, Scott A Rutherford, Andrew T King, Charlotte L Hammerbeck-Ward, Omar Pathmanaban, Helen Entwistle, Judith Bird, Patrick R Axon, David A Moffat, Simon K Lloyd

**Affiliations:** 1 Brighton and Sussex Medical School, Brighton, UK; 2 MOH Holdings, Singapore; 3 Department of Neurosurgery, Manchester Academic Health Science Centre, Salford Royal NHS Foundation Trust, Manchester, UK; 4 Department of Otolaryngology, Manchester Academic Health Science Centre, Salford Royal NHS Foundation Trust, Manchester, UK; 5 Geoffrey Jefferson Brain Research Centre, Manchester Academic Health Science Centre, Manchester, UK; 6 Division of Cardiovascular Sciences, School of Medical Sciences, Faculty of Brain and Mental Health, University of Manchester, Manchester, UK; 7 Division of Neuroscience and Experimental Psychology, School of Biological Sciences, Faculty of Brain and Mental Health, University of Manchester, Manchester, UK; 8 Department of Otolaryngology, Addenbrooke's Hospital, Cambridge, UK; 9 Division of Cancer Sciences, School of Medical Sciences, University of Manchester, Manchester, UK

**Keywords:** Acoustic neuroma, sensorineural hearing loss, audiology, skull base, surgery, facial nerve, tinnitus

## Abstract

**Objective:**

This study aimed to assess degree of audiovestibular handicap in patients with vestibular schwannoma.

**Methods:**

Audiovestibular handicap was assessed using the Hearing Handicap Inventory, Tinnitus Handicap Inventory and Dizziness Handicap Inventory. Patients completed questionnaires at presentation and at least one year following treatment with microsurgery, stereotactic radiosurgery or observation. Changes in audiovestibular handicap and factors affecting audiovestibular handicap were assessed.

**Results:**

All handicap scores increased at follow up, but not significantly. The Tinnitus Handicap Inventory and Dizziness Handicap Inventory scores predicted tinnitus and dizziness respectively. The Hearing Handicap Inventory was not predictive of hearing loss. Age predicted Tinnitus Handicap Inventory score and microsurgery was associated with a deterioration in Dizziness Handicap Inventory score.

**Conclusion:**

Audiovestibular handicap is common in patients with vestibular schwannoma, with 75 per cent having some degree of handicap in at least one inventory. The overall burden of handicap was, however, low. The increased audiovestibular handicap over time was not statistically significant, irrespective of treatment modality.

## Introduction

Vestibular schwannomas are benign, slow-growing tumours that arise on the VIIIth cranial nerve. They originate from the Schwann cells of the vestibular portion of the vestibulocochlear nerve and account for about 6 per cent of all intracranial tumours.^1^ They usually present with audiovestibular symptoms, including hearing loss (97 per cent), tinnitus (70 per cent) and imbalance (46 per cent).^2^ Vestibular schwannoma can also cause other cranial nerve symptoms including facial numbness and occasionally facial paralysis.^2,3^ Large tumours causing cerebral compression can lead to ataxia, cerebellar tonsil herniation, hydrocephalus and eventually death if left untreated,^2^ although this is unusual in modern clinical practice.

There are three treatment options: microsurgery, radiotherapy – usually in the form of stereotactic radiosurgery, and observation (watch, wait and rescan). Decision-making around choice of treatment is complex, and takes into consideration patient age, tumour size, symptoms, the presence of co-morbidities, local expertise and patient preference.

Handicap arising from the symptoms of vestibular schwannoma or their treatment can have a significant impact on the patient's quality of life, with audiovestibular symptoms being particularly influential.^2^

There are a number of ways that audiovestibular handicap can be quantified, but the audiovestibular handicap inventories (Hearing Handicap Inventory, Tinnitus Handicap Inventory and Dizziness Handicap Inventory) have been widely used and well validated.^4–6^

Most of the literature related to audiovestibular handicap in patients with vestibular schwannoma provides cross-sectional data for patients undergoing active treatment. Very few studies have investigated audiovestibular handicap in patients undergoing observation, and fewer still have investigated how the handicap changes over time across modalities. This paper addresses these deficiencies.

## Materials and methods

The study was approved by the National Research and Ethics Service (approval code: 17/EM0261). Patients aged over 18 years with a sporadic vestibular schwannoma smaller than 3 cm (defined below), who had been followed up for at least 1 year following diagnosis and treatment, were included. Patients with neurofibromatosis type 2, those who had tumours over 3 cm in size and those who had received more than one modality of active treatment were excluded. Tumour size was limited to less than 3 cm because patients with tumours below this size are unlikely to have non-audiovestibular complications that would otherwise prove to be confounding factors.

There were two cohorts of patients included in the study. Cohort one was managed at Salford Royal Hospital. These patients were asked to complete the three audiovestibular handicap inventories when they initially presented, prior to any treatment. They then went on to have one of the three treatment modalities. They were posted a second set of audiovestibular handicap inventories at least one year after treatment which they subsequently returned.

The second cohort of patients, managed at Addenbrooke's Hospital, only included patients with untreated sporadic tumours undergoing observation. This cohort did not complete questionnaires at presentation. They were invited to a research clinic, where they completed the audiovestibular handicap questionnaires and underwent pure tone audiometry and speech audiometry using Arthur Boothroyd word lists. Patients’ hearing was categorised according to recommendations made by the American Academy of Otolaryngology – Head and Neck Surgery, and graded from class A to D.^7^ As per these recommendations, pure tone averages were calculated using thresholds at 0.5, 1, 2 and 3 kHz. Speech discrimination at 40 dB sensation level (i.e. 40 dB above the threshold or maximum comfortable loudness, whichever was less) was recorded using Arthur Boothroyd word lists.

Other data collected included patient demographics (age at diagnosis, gender), symptoms (particularly hearing loss, tinnitus and dizziness) and tumour characteristics (tumour side, and size before and at the time of treatment). The presence of audiovestibular symptoms was determined through subjective reporting by the patients. Subjective severity of tinnitus and dizziness was not recorded.

Tumour size was measured using the cerebellopontine angle component with intracanalicular tumours measuring 0 mm. The measurement used was the distance from the porous to the most medial portion of the tumour, down a line running along the middle of the long axis of the internal auditory meatus.

### Audiovestibular handicap questionnaires

Each of the handicap inventories consists of 25 questions encompassing different components of potential handicap related to a particular audiovestibular symptom. The questions may be answered ‘yes’, ‘sometimes’ or ‘no’, scoring ‘4’, ‘2’ or ‘0’, respectively. This generates a score from 0 to 100. A higher score indicates greater handicap. Each inventory divides its score into degrees of severity from none through mild to severe.

### Statistical analysis

Statistical analysis was carried out using SPSS® Statistics software (version 23.0.0.0). Spearman's correlation test was performed to assess the relationship between audiovestibular handicap and categorical data. Pearson's correlation test was performed to assess the relationship between audiovestibular handicap and continuous data. Change in audiovestibular handicap was analysed using the Kruskal–Wallis test.

A multiple linear regression was performed to identify potential predictors of audiovestibular handicap scores. The factors included were: patient's gender, age, side of tumour, tumour size at treatment and presence of the relevant symptom at presentation. Treatment modality was also included as a variable in the regression model for those patients managed at Salford Royal Hospital, in order to determine if the different treatments influenced the change in pre- and post-treatment audiovestibular handicap scores. Statistical significance was set as *p* < 0.05.

### Patients

A total of 108 patients from Salford Royal Hospital were invited to take part. Of these, 65 patients returned the follow-up questionnaires (return rate of 60 per cent). Four questionnaires were returned incomplete, leaving a cohort of 61 patients.

Twenty-three of the 61 patients received stereotactic radiosurgery, 21 had microsurgery and 17 opted for observation. The mean age at presentation was 61 years (range, 31–79 years; standard deviation (SD) = 11.1). Those undergoing stereotactic radiosurgery had the oldest mean age at treatment (64.1 years; range, 47–76 years; SD = 8.3). Those undergoing microsurgery had the youngest mean age at treatment (56.6 years; range, 31–74 years; SD = 12.7). There were 29 males and 32 females.

The mean tumour size at diagnosis was 8.07 mm (SD = 6.8). The mean tumour size at presentation for those patients undergoing observation, microsurgery or stereotactic radiosurgery was 4.7 mm (range, 0–17 mm; SD = 5.5), 14.2 mm (range, 4–30 mm; SD = 8.8) and 5.3 mm (range, 5–13 mm; SD = 4.3), respectively. The differences in tumour size between groups was statistically significant. The mean tumour size at treatment for those patients undergoing stereotactic radiosurgery was 9.8 mm (range, 7–21 mm; SD = 5.6), compared to 17.5 mm (range, 7–30 mm; SD = 8.1) for those undergoing microsurgery. There were 36 left-sided tumours (59 per cent) and 25 right-sided tumours (41 per cent).

For observation, microsurgery and stereotactic radiosurgery, the mean follow-up duration was 43 months (range, 24–52 months; SD = 7.8), 30.9 months (range, 12–58 months; SD = 13.2) and 33.6 months (range, 13–48 months; SD = 11.9), respectively.

A total of 257 patients from Addenbrooke's Hospital were invited to take part. Of these, 179 patients completed the audiovestibular handicap questionnaires (return rate of 70 per cent). The mean age at presentation was 60.2 years (range, 26–91 years; SD = 11.4). There were 96 males (53.6 per cent) and 83 females (46.4 per cent). The mean tumour size at diagnosis was 9.0 mm (range, 0–30 mm; SD = 4.2). There were 78 left-sided tumours (43.6 per cent) and 101 right-sided tumours (56.4 per cent). The mean follow-up duration was 72.5 months (range, 16.9–224.2 months; SD = 37.3).

The mean age of those who declined to take part or who did not return questionnaires was greater than that of patients who agreed to take part (72.1 *vs* 66.6; *p* = 0.02). Similarly, the female-to-male ratio was greater in the group who agreed to take part (0.9 *vs* 0.7; *p* = 0.05). There was no difference in tumour size between those who agreed and those who declined to take part.

## Results

### Symptoms at presentation

A total of 227 patients presented with hearing loss (93 per cent), 111 had dizziness or imbalance (45.5 per cent), and 169 had tinnitus (69.3 per cent).

Of the Salford Royal Hospital cohort, 15 out of 17 patients (88.2 per cent) who were being managed conservatively (watch, wait and rescan group), 20 of 23 (87 per cent) undergoing stereotactic radiosurgery and 18 of 21 (85.8 per cent) undergoing microsurgery had hearing loss on presentation. Five patients (29.4 per cent) who were conservatively managed, 12 (52.2 per cent) who underwent stereotactic radiosurgery and 9 (42.9 per cent) who underwent microsurgery presented with dizziness. Ten patients (58.8 per cent) in the conservative management group, 17 (73.9 per cent) in the stereotactic radiosurgery group and 14 (66.7 per cent) in the microsurgery group presented with tinnitus. Only six patients in the Salford cohort as a whole presented with a headache (9.8 per cent): two (11.8 per cent) in the watch, wait and rescan group, and four (19.0 per cent) in the microsurgery group. At presentation, three patients had facial weakness and four had facial numbness.

### Audiology

In the Addenbrookes group, the mean pure tone audiometric average was 57.3 dB (SD = 26.3) in the ear containing the tumour (ipsilateral ear) and 25.0 dB (SD = 17.8) in the opposite ear (contralateral ear).

Table 1 summarises the American Academy of Otolaryngology – Head and Neck Surgery hearing class for the Addenbrooke's patients at the time of completion of the audiovestibular handicap questionnaires. In the ipsilateral ear, the mean speech discrimination score at 40 dB was 23.1 per cent (SD = 31.8) and the mean maximum speech discrimination score was 57.9 per cent (SD = 35.0). In the contralateral ear at review, the mean speech discrimination score at 40 dB was 66.0 per cent (SD = 34.9) and the mean maximum speech discrimination score was 93.0 per cent (SD = 16.0).

**Table 1.** Distribution of AAO-HNS hearing class in ipsilateral and contralateral ears

Side	AAO-HNS class	Cases (*n* (%))
Ipsilateral	A	16 (13.0)
	B	14 (10.6)
	C	1 (0.8)
	D	98 (75.6)
Contralateral	A	78 (60.2)
	B	13 (10.6)
	C	–
	D	38 (29.3)

AAO-HNS = American Academy of Otolaryngology – Head and Neck Surgery

### Initial audiovestibular handicap by treatment modality

#### Observation

**Figure 1.**
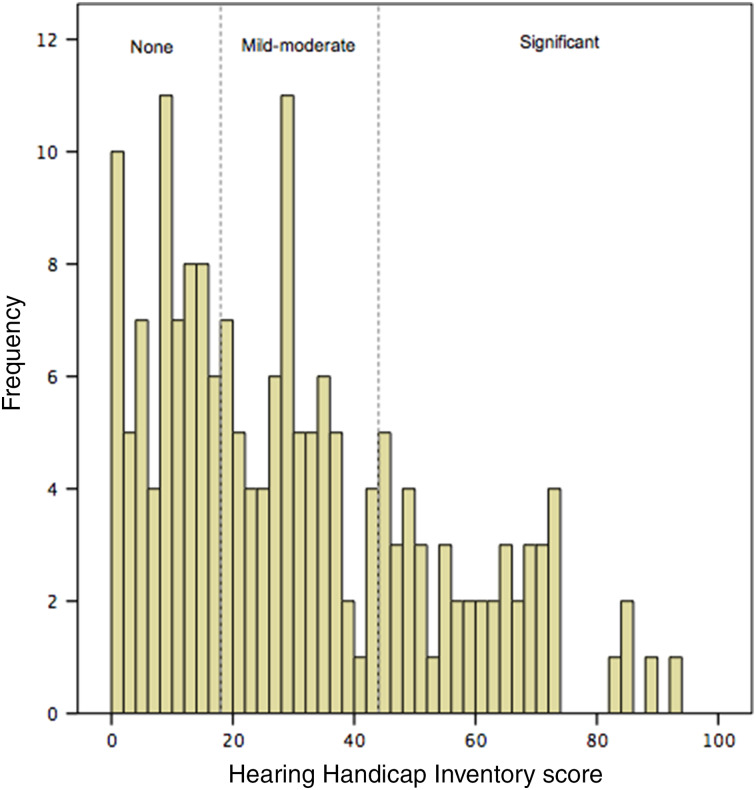
Histogram showing the distribution of Hearing Handicap Inventory scores. The scores are divided according to severity.

Of the 196 patients who had no active treatment, the mean Hearing Handicap Inventory score was 30.0 (range, 0–96; SD = 23.1). Thirty-seven per cent had no hearing handicap (scores of 0–16), 35 per cent had mild to moderate hearing handicap (scores of 17–42), and 28 per cent had severe hearing handicap (scores over 42). The distribution of Hearing Handicap Inventory scores is shown in Figure 1.

**Figure 2.**
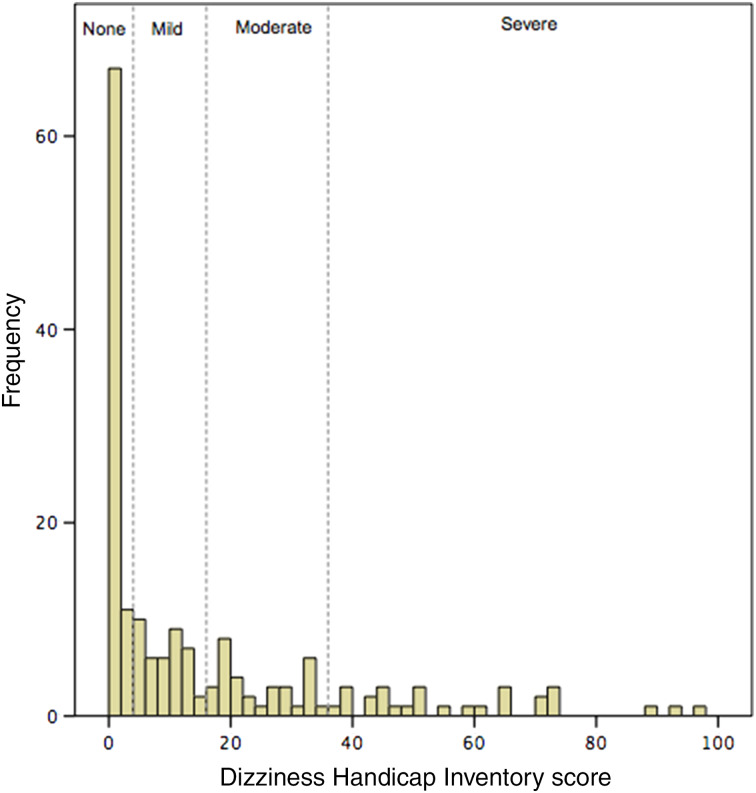
Histogram showing the distribution of Dizziness Handicap Inventory scores. The scores are divided according to severity.

The mean Dizziness Handicap Inventory score for this group was 16.0 (range, 0–96; SD = 21.1). Thirty-seven per cent had no dizziness handicap (scores of 0–2), 26 per cent had mild dizziness handicap (scores of 3–14), 18 per cent had moderate dizziness handicap (scores of 15–34), and 18 per cent had severe dizziness handicap (scores over 34). Of those who had some dizziness handicap, 60 per cent were moderately or severely affected. The distribution of Dizziness Handicap Inventory scores is shown in Figure 2.

**Figure 3.**
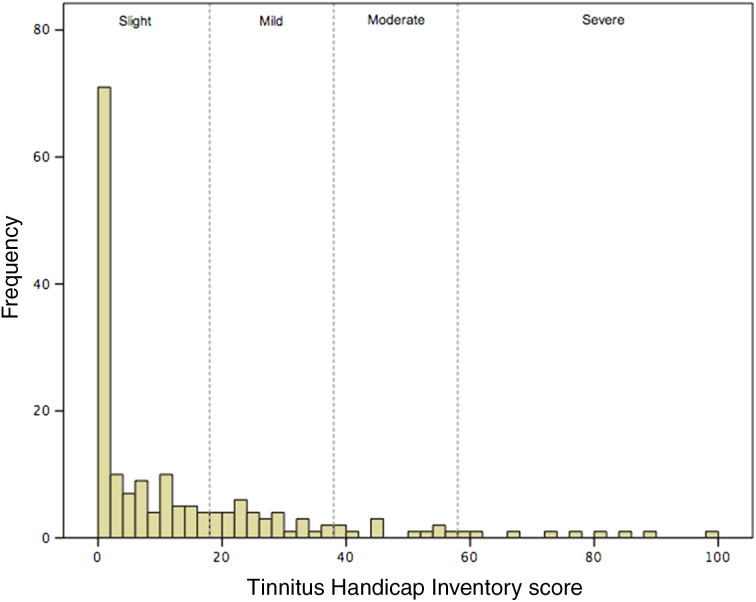
Histogram showing the distribution of Tinnitus Handicap Inventory scores. The scores are divided according to severity.

The mean Tinnitus Handicap Inventory score for this group was 14.3 (range, 0–98; SD = 20.5). Seventy per cent had slight tinnitus handicap (scores of 0–16), 18 per cent had mild tinnitus handicap (scores of 17–36), 6 per cent had moderate tinnitus handicap (scores of 37–56) and 6 per cent had severe tinnitus handicap (scores over 56). The distribution of Tinnitus Handicap Inventory scores is shown in Figure 3.

Of the patients, 77.8 per cent had mild to severe handicap in at least one inventory, 48.9 per cent had mild to severe handicap in at least two inventories, and 23.3 per cent had mild to severe handicap in all three inventories; 2.8 per cent of the patients had severe handicap in all three inventories.

#### Microsurgery

Of the 21 Salford patients who underwent microsurgery, the mean Hearing Handicap Inventory score was 43.1 at presentation. Nineteen per cent had no hearing handicap (scores of 0–16), 19 per cent had mild to moderate hearing handicap (scores of 17–42), and 62 per cent had severe hearing handicap (scores over 42).

The mean Dizziness Handicap Inventory score was 26.2 at presentation (range, 0–60; SD = 21.1). Nineteen per cent had no dizziness handicap (scores of 0–2), 24 per cent had mild dizziness handicap (scores of 3–14), 19 per cent had moderate dizziness handicap (scores of 15–34), and 38 per cent had severe dizziness handicap (scores over 34).

The mean Tinnitus Handicap Inventory score was 22.9 at presentation (range, 0–96; SD 28.9). Fifty-seven per cent had slight tinnitus handicap (scores of 0–16), 24 per cent had mild tinnitus handicap (scores of 17–36), 0 per cent had moderate tinnitus handicap (scores of 37–56) and 19 per cent had severe tinnitus handicap (scores over 56).

#### Stereotactic radiosurgery

Of the 23 Salford cohort of patients who underwent stereotactic radiosurgery, the mean Hearing Handicap Inventory score was 29.1 at presentation. Forty-three per cent had no hearing handicap (scores of 0–16), 22 per cent had mild to moderate hearing handicap (scores of 17–42), and 35 per cent had severe hearing handicap (scores over 42).

The mean Dizziness Handicap Inventory score was 16.2 at presentation (range, 0–70; SD = 21.1). Thirty-nine per cent had no dizziness handicap (scores of 0–2), 22 per cent had mild dizziness handicap (scores of 3–14), 17 per cent had moderate dizziness handicap (scores of 15–34), and 22 per cent had severe dizziness handicap (scores over 34).

The mean Tinnitus Handicap Inventory score was 10 at presentation (range, 0–54; SD = 14.5). Seventy-eight per cent had slight tinnitus handicap (scores of 0–16), 17 per cent had mild tinnitus handicap (scores 17–36), 5 per cent had moderate tinnitus handicap (scores of 37–56) and 0 per cent had severe tinnitus handicap (scores over 56).

### Changes in audiovestibular handicap over time

The pre- and post-treatment handicap inventory scores for the three treatment modalities are shown in Table 2. All handicap inventory scores increased (worsened) after treatment, with the exception of Tinnitus Handicap Inventory score, which decreased in the group undergoing observation. These changes were not, however, statistically significant.

**Table 2.** Pre- and post-treatment audiovestibular handicap scores for each treatment modalities

Treatment modality	HHI scores		DHI scores		THI scores	
	Pre	Post	Pre	Post	Pre	Post
Watch, wait & rescan (*n* = 17)						
– Mean	23.8	33.9	12.0	15.5	15.6	14.7
– SD	20.6	19.0	20.0	19.0	22.6	16.8
– Range	0–84	0–54	0–72	0–54	0–80	0–58
Stereotactic radiosurgery (*n* = 23)						
– Mean	29.1	31.2	16.2	21.5	10.0	12.5
– SD	24.2	25.3	21.1	25.1	14.5	17.9
– Range	0–72	0–94	0–70	0–74	0–54	0–60
Microsurgery (*n* = 21)						
– Mean	43.1	49.9	26.2	42.0	22.9	25.4
– SD	27.7	28.4	21.1	29.6	28.9	29.1
– Range	0–96	0–98	0–54	0–92	0–96	0–100
Total (*n* = 61)						
– Mean	32.5	38.4	18.5	26.9	16.0	17.6
– SD	25.5	27.5	21.3	27.3	22.8	22.5
– Range	0–96	0–98	0–72	0–92	0–96	0–100

HHI = Hearing Handicap Inventory; DHI = Dizziness Handicap Inventory; THI = Tinnitus Handicap Inventory; SD = standard deviation

### Predictors of audiovestibular handicap score

Scores for all three handicap inventories were statistically significantly correlated, although weakly (Hearing Handicap Inventory *vs* Tinnitus Handicap Inventory, *p* < 0.0001 and r = 0.269; Hearing Handicap Inventory *vs* Dizziness Handicap Inventory, *p* < 0.0001 and r = 0.304; Tinnitus Handicap Inventory *vs* Dizziness Handicap Inventory, *p* < 0.0001 and r = 0.338 (Kendall's tau coefficient)).

#### Hearing handicap

**Figure 4.**
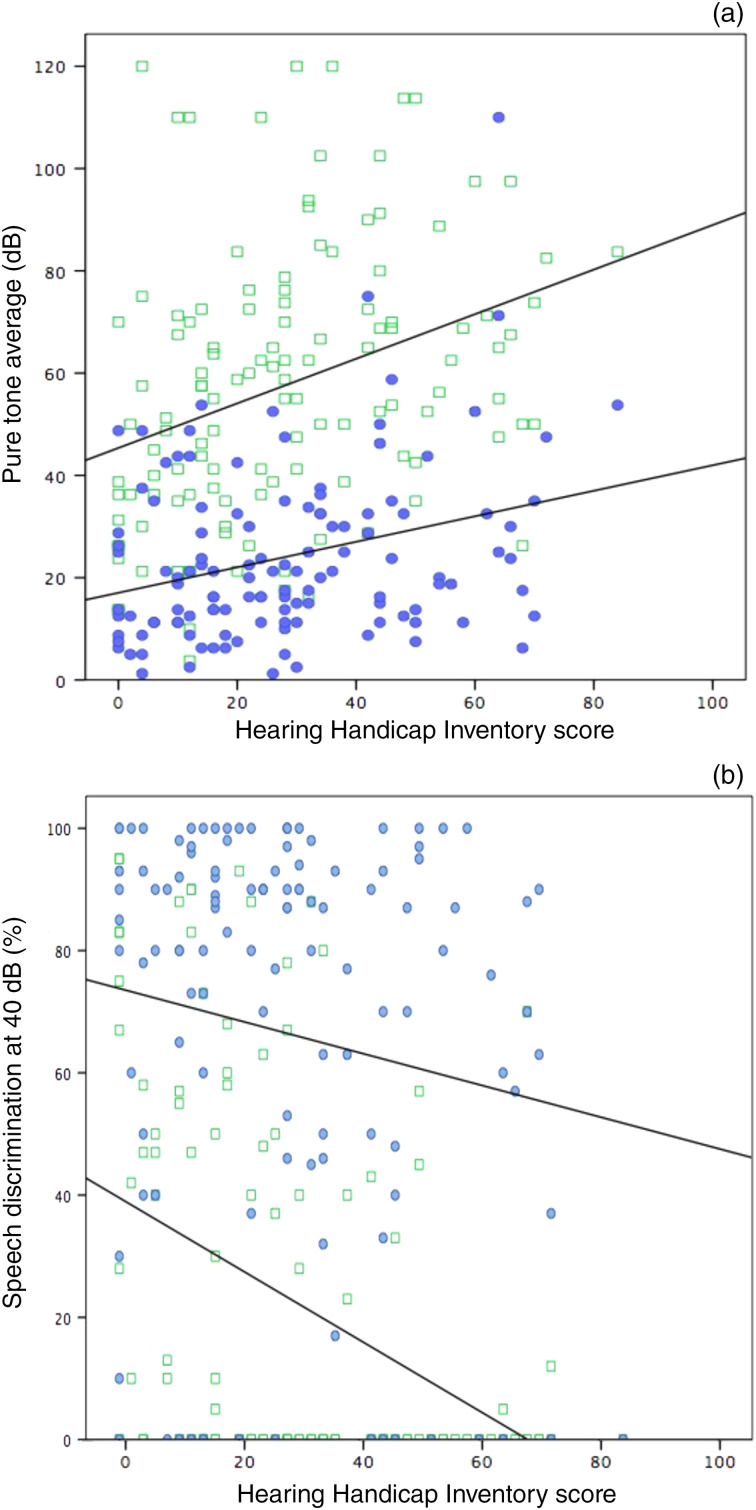
Scatterplot illustrating the correlation between Hearing Handicap Inventory scores and: (a) pure tone average and (b) speech discrimination scores. The blue dots represent the contralateral ear and the green squares represent the ipsilateral ear. The lines of best fit for the contralateral and ipsilateral ears are shown.

There was a weak correlation between the presence of hearing loss and Hearing Handicap Inventory score (r = 0.128 and *p* = 0.046), and a weak correlation between the presence of dizziness and Hearing Handicap Inventory score (r = 0.251 and *p* < 0.001). There was no correlation between the presence of tinnitus and Hearing Handicap Inventory score. There was a significant although weak correlation between Hearing Handicap Inventory score and mean ipsilateral pure tone audiological average (*p* < 0.0001 and r = 0.260; Kendall's tau coefficient). There was also a significant although weak correlation between Hearing Handicap Inventory score and contralateral pure tone audiological average (*p* = 0.001 and r = 0.202; Kendall's tau coefficient). This is illustrated in Figure 4a.

There were significant but weak correlations between Hearing Handicap Inventory score and ipsilateral speech discrimination score at 40 dB (*p* < 0.0001 and r = −0.302; Kendall's tau coefficient) and maximum speech discrimination (*p* = 0.001 and r = −0.205; Kendall's tau coefficient). There was also a significant although weak negative correlation between Hearing Handicap Inventory score and contralateral maximum speech discrimination score (*p* = 0.024 and r = −0.157; Kendall's tau coefficient), but no significant correlation with contralateral speech discrimination at 40 dB (*p* = 0.124 and r = −0.098; Kendall's tau coefficient). The correlation between Hearing Handicap Inventory scores and speech discrimination at 40 dB is shown in Figure 4b.

#### Dizziness handicap

There was a moderate correlation between the presence of dizziness and Dizziness Handicap Inventory score (r = 0.661 and *p* < 0.001), but no correlation between the presence of hearing loss or tinnitus and Dizziness Handicap Inventory score. There was a statistically significant difference in Dizziness Handicap Inventory score between male and female gender (*p* = 0.13; Mann–Whitney U test). No significant association was found between Dizziness Handicap Inventory score and age (*p* = 0.845 and r = 0.011; Kendall's tau coefficient) or tumour size (*p* = 0.138 and r = 0.081; Kendall's tau coefficient).

#### Tinnitus handicap

There was a moderate correlation between the presence of tinnitus and Tinnitus Handicap Inventory score (r = 0.561 and *p* < 0.001), and a weak correlation between presence of dizziness and Tinnitus Handicap Inventory score (r = 0.251 and *p* < 0.001). There was no correlation between the presence of hearing loss and Tinnitus Handicap Inventory score. There was no significant correlation between Tinnitus Handicap Inventory score and mean ipsilateral pure tone audiological average (*p* = 0.272 and r = −0.070; Kendall's tau coefficient) or contralateral pure tone audiological average (*p* = 0.515 and r = −0.042; Kendall's tau coefficient). Similarly, there was no correlation between Tinnitus Handicap Inventory score and ipsilateral speech discrimination at 40 dB (*p* = 528 and r = 0.045; Kendall's tau coefficient), ipsilateral maximum speech discrimination (*p* = 0.145 and r = 0.097; Kendall's tau coefficient), contralateral speech discrimination at 40 dB (*p* = 0.905 and r = 0.008; Kendall's tau coefficient) or contralateral maximum speech discrimination (*p* = 0.366 and r = 0.066; Kendall's tau coefficient).

Severe tinnitus handicap was strongly associated with severe handicap in the other inventories. Of the nine patients with severe tinnitus, 77.8 per cent also had severe imbalance and 77.8 per cent also had significant hearing handicap. This compares to 25 per cent and 67.9 per cent of patients with severe dizziness handicap having severe tinnitus handicap and significant hearing handicap respectively. It also compares to 14.9 per cent and 67.9 per cent of patients with significant hearing handicap who had severe tinnitus and dizziness handicap respectively.

The multiple linear regression analysis revealed no significant predictors of Hearing Handicap Inventory score. The presence of dizziness was found to be a significant predictor of Dizziness Handicap Inventory score (*p* < 0.001), and tinnitus was found to be a significant predictor of Tinnitus Handicap Inventory score (*p* < 0.001). Age was also found to be a significant predictor of Tinnitus Handicap Inventory (*p* = 0.044). Microsurgery was shown to have a statistical significance in worsening Dizziness Handicap Inventory when compared with observation (*p* = 0.033) and stereotactic radiosurgery (*p* = 0.044). No other demographic or tumour factors had a significant effect on handicap scores.

## Discussion

This study shows that audiovestibular handicap is common amongst patients with sporadic vestibular schwannoma, irrespective of treatment modality, with over 75 per cent having some degree of handicap in at least one inventory. This is a similar finding to that of other authors.^8,9^ The relationship between audiovestibular symptoms and handicap was, however, complex. Although 93 per cent of patients described subjective hearing loss, 37 per cent had no hearing disability arising from it. This may reflect the fact that the ipsilateral hearing loss was mild in some cases, but probably also reflects the presence of good hearing in the contralateral ear. The loss of directional sound and the reduced ability to hear in noise – two issues that arise when an individual has a unilateral hearing loss – may not have much impact on day-to-day hearing disability. It is also possible that the Hearing Handicap Inventory is not sensitive enough to identify subtle disabilities.

The overall burden of handicap due to dizziness at presentation was relatively low, with a mean Dizziness Handicap Inventory score of 16. Although 41.3 per cent of patients experienced dizziness, 63 per cent of the population had either no handicap or mild handicap. If dizziness was present, however, 60 per cent were moderately or severely handicapped by it. The presence of dizziness has been shown to be the most important predictor of quality of life in patients with vestibular schwannoma.^3,10^

Tinnitus was more common than dizziness, but again the overall burden of disability was low. The mean handicap score was 14.3, and 88 per cent of patients had slight (which includes no tinnitus) or mild tinnitus handicap. Unlike dizziness, if tinnitus was present, only 12 per cent had moderate or severe handicap arising from it.

It is important to note that there is a degree of handicap due to hearing impairment, dizziness and tinnitus in the normal population, particularly in the age group affected by vestibular schwannomas; given that there was no control group in this study, it is not possible to quantify how much of this handicap was due to the presence of a vestibular schwannoma.

The absence of significant change in any of the handicap inventories following all modalities of treatment was interesting. The literature is conflicting with regard to the effect of microsurgery and stereotactic radiosurgery on tinnitus. Some suggest that microsurgery reduces tinnitus.^11^ Others suggest minimal overall change.^11^ Similarly, stereotactic radiosurgery increases tinnitus in some series,^11^ but it is generally accepted as having a minimal overall effect.^8,12–14^ The literature investigating the effects of microsurgery and stereotactic radiosurgery on balance is also inconsistent. Microsurgery has historically been offered as a means of improving balance disturbance in those who are handicapped by it. The results of this study suggest that surgery does not result in significant improvement in dizziness handicap and, in fact, suggests the opposite. The cohort was, however, relatively small and the results need to be interpreted with care. This finding is, however, consistent with the results of a recent systematic review by Ojha and Clamp, which suggested that surgery may not have a useful role in improving balance function.^15^ Stereotactic radiosurgery appears to have subtle detrimental effects on balance function in some series,^13^ but improves balance function in others.^14^

The lack of change in hearing handicap over time is also surprising. This may be because a significant proportion of hearing is already lost by the time the patient presents, so any disability will already have arisen. Further hearing loss occurring over time may therefore not result in significant additional disability. Humphriss *et al*.^16^ have shown that the better the hearing class at presentation, the greater the change in hearing handicap over time, which suggests it is only those who have good hearing that notice increased handicap. It may also be that unilateral hearing loss does not cause significant disability, or perhaps the tool used is not sensitive enough to measure the types of hearing disability that might arise from further unilateral hearing loss.

- Handicaps arising from vestibular schwannoma symptoms or their treatment can significantly affect quality of life
- Dizziness, tinnitus and hearing handicaps are quantified in their respective handicap inventory scores, which are widely use and well validated
- Most of the literature around audiovestibular handicaps does not include patients undergoing observation as opposed to active treatment
- Previous research also does not address how handicap changes over time across various treatment modalities
- Audiovestibular handicap is common following diagnosis and/or treatment of a vestibular schwannoma
- However, the burden is low, and does not significantly increase over time, irrespective of treatment modality

### Predictors of audiovestibular scores

The absence of correlation between certain demographic and tumour-related parameters and handicap has been widely noted elsewhere. Jufas *et al*. and Lloyd *et al*. have both reported an absence of association with gender and audiovestibular handicap,^3,17^ although Humphriss *et al*. found that female gender was associated with higher Dizziness Handicap Inventory scores.^16^ Similarly, Wagner *et al*. and Lloyd *et al*. showed no association between tumour size and audiovestibular handicap.^3,18^ The association between age and tinnitus handicap has, however, been noted by others,^18,19^ although not universally.^18^ This may not be directly related to the presence of the vestibular schwannoma, as tinnitus is more common with age. Idiopathic tinnitus is also reported to be more severe in the elderly population.^20,21^

### Study limitations

The absence of a control group makes it impossible to know how much handicap was related to the vestibular schwannoma rather than other factors that affect the general population. The number of patients in the treatment groups of the Salford Royal Hospital cohort was also small. It was also impossible to accurately match patients across the treatment groups in the Salford cohort, particularly because of fundamental differences in tumour size (tumours treated surgically tend to be larger) and certain demographics (patients opting for stereotactic radiosurgery tend to be older). It was also not possible to correlate subjective severity of dizziness and tinnitus with dizziness and tinnitus handicap, because of the limitations in the dataset collected. Finally, there is likely to be selection bias resulting from differences between those patients who responded to the invitation to take part in the study and those who did not. For example, older patients were less likely to respond than younger patients.

## Conclusion

This study provides new data related to audiovestibular handicap in patients undergoing observation, and is unique in investigating changes in handicap over time across all treatment modalities. Whilst audiovestibular handicap is common amongst patients with vestibular schwannoma, the relationship between the presence of a symptom and the degree of handicap arising from it is complex, with the Tinnitus Handicap Inventory and Dizziness Handicap Inventory potentially being better measures of handicap than the Hearing Handicap Inventory. Treatment appears not to have a significant influence on audiovestibular handicap.
